# Immunogenicity, Effectiveness, and Safety of COVID-19 Vaccines in Rheumatic Patients: An Updated Systematic Review and Meta-Analysis

**DOI:** 10.3390/biomedicines10040834

**Published:** 2022-04-01

**Authors:** Kuo-Tung Tang, Bo-Chueh Hsu, Der-Yuan Chen

**Affiliations:** 1 Division of Allergy, Immunology and Rheumatology, Taichung Veterans General Hospital, Taichung 407, Taiwan; dirac1982@vghtc.gov.tw; 2 School of Medicine, National Yang Ming Chiao Tung University, Taipei 112, Taiwan; 3 Ph.D. Program in Translational Medicine, Rong Hsing Research Center for Translational Medicine, National Chung Hsing University, Taichung 402, Taiwan; 4 Division of Allergy, Immunology and Rheumatology, Taichung Veterans General Hospital Puli Branch, Nantou 545, Taiwan; vhpld0273@vghtc.gov.tw; 5 College of Medicine, China Medical University, Taichung 404, Taiwan; 6 Translational Medicine Laboratory, Rheumatology and Immunology Center, China Medical University Hospital, Taichung 404, Taiwan

**Keywords:** adverse events, COVID-19 vaccine, flare, immunogenicity, rheumatic disease, safety

## Abstract

Background: Vaccination is one of the most important measures worldwide to halt the spread of the corona virus disease 2019 (COVID-19). However, the efficacy and safety of these vaccines in rheumatic patients are not well explored. Therefore, we conducted a systematic review and meta-analysis. Methods: We performed a literature search of the PubMed and EMBASE databases on 17 November 2021. Forty-seven studies relevant to the immunogenicity, efficacy/effectiveness, and safety of COVID-19 vaccines were selected. Results: Our results demonstrated that COVID-19 vaccination is effective in protecting rheumatic patients from severe illness caused by the virus. Both the humoral and cellular immunogenicity of vaccines were impaired in rheumatic patients, which were greatly enhanced after the second vaccine dose. Receiving anti-CD20 therapy was associated with impaired humoral immunogenicity. Adverse events due to COVID-19 vaccines in rheumatic patients were similar to those in healthy controls, except for an increased incidence of arthralgia. The incidence of disease flares after COVID-19 vaccination was low. Conclusion: Our systematic review indicated the importance of full vaccination in rheumatic patients. Withholding anti-CD20 therapy was found to be potentially beneficial for the immunogenicity. Furthermore, the vaccines were found to be safe in general. Despite significant heterogeneity between studies, we recommend that rheumatic patients receive these vaccines amidst the global pandemic.

## 1. Introduction

Since the initial outbreak in December 2019, the coronavirus disease 2019 (COVID-19) pandemic has placed a tremendous burden on healthcare systems and is still a huge threat to all human beings. As of 31 December 2021, nearly 285 million cases have been diagnosed, and 5.1 million fatalities reported globally. The severity of the disease may be alleviated by the global application of effective and safe vaccinations [1]. The mRNA and recombinant adenovirus formats are novel vaccine technologies; however, only healthy or immunocompetent adults were systemically assessed for immunogenicity and the safety of COVID-19 vaccines in phase I, II, and III clinical trials [2,3,4,5,6,7,8,9,10]. To date, very few phase IV clinical trials have been conducted to evaluate the immunogenicity and safety of vaccines for patients with rheumatic diseases [11,12,13,14].

Increasing evidence indicates that COVID-19 poses a higher risk for rheumatic patients of more severe disease and mortality, which include systemic lupus erythematosus (SLE), rheumatoid arthritis (RA), systemic sclerosis, and idiopathic inflammatory myositis, compared with healthy individuals [15,16,17]. However, the safety profile of COVID-19 vaccines has been relatively unexplored in rheumatic patients, and multiple studies have demonstrated that the immunogenicity of vaccines may be attenuated by the use of certain immunosuppressants or biologics [13,18,19,20,21,22]. While making efforts in terms of widespread vaccination, physicians also need to understand the immunogenicity and adverse effects of vaccines in such patients, including the possibility of vaccine-induced exacerbation of pre-existing rheumatic diseases. Therefore, we aim to update the evidence of immunogenicity, efficacy/effectiveness, and safety of COVID-19 vaccines in rheumatic patients. Hopefully, our results will have beneficial clinical implications for these vulnerable populations.

## 2. Materials and Methods

### 2.1. Literature Search

**Figure 1:**
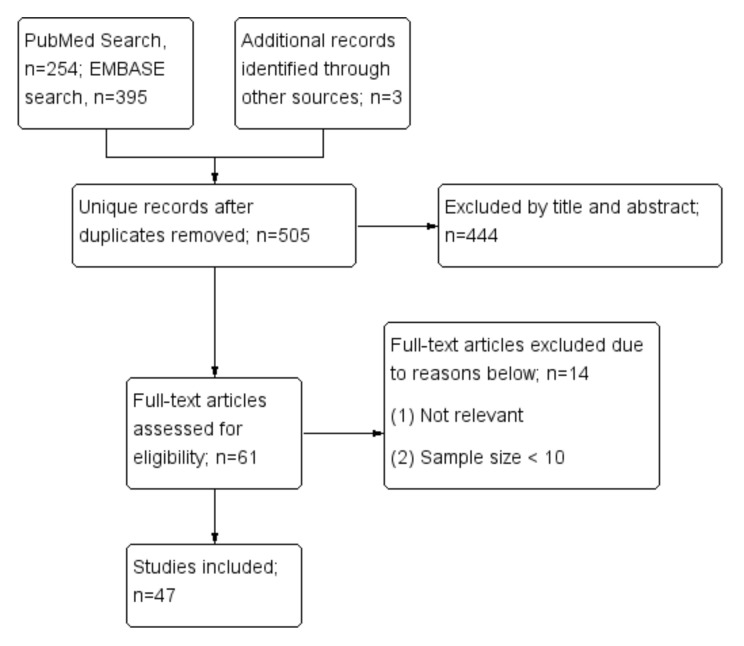
Flow diagram based on the Preferred Reporting Items for Systematic Reviews and Meta-Analyses (PRISMA) guidelines.

The present review focuses on existing evidence of the immunogenicity and safety profile of COVID-19 vaccines in rheumatic patients. The algorithm of the systematic review followed the Preferred Reporting Items for Systematic Reviews and Meta-Analyses (PRISMA) checklist. We searched the EMBASE and MEDLINE databases. We reviewed English literature from 1 January 2020 to 17 November 2021. The search keywords for COVID-19 vaccines included those on the World Health Organization (WHO) list: mRNA-1273 (Moderna, Cambridge, MA, USA), BNT162b2 (Pfizer–BioNTech, Mainz, Germany), Ad26.COV2.S. (Johnson & Johnson–Janssen, New Brunswick, NJ, USA), AZD1222 (AstraZeneca, Cambridge, UK), Covaxin (Bharat Biotech, Hyderabad, India), BBIBP-CorV (Sinopharm, Beijing, China) and CoronaVac (Sinovac, Beijing, China). The keywords for rheumatic diseases include inflammatory arthritis, SLE, Sjogren’s syndrome, systemic sclerosis, idiopathic inflammatory myositis, antiphospholipid syndrome, vasculitis, cryoglobulinemia, adult-onset Still’s disease, and fibromyalgia. The details of the search strategy are illustrated in Supplementary Table S1. Eventually, we identified a total of 47 studies according to the Preferred Reporting Items for Systematic Reviews and Meta-Analyses (PRISMA) guidelines (Figure 1). The present study has been registered in PROSPERO (CRD42022307795).

### 2.2. Study Selection

Three authors (KT Tang, BC Hsu, and DY Chen) independently assessed the titles and abstracts identified by the search mentioned above and retrieved the relevant full-text articles. Two authors (KT Tang and DY Chen) independently evaluated the full-text articles for eligibility. We selected potentially relevant articles on immunogenicity, efficacy/effectiveness, and/or safety of COVID-19 vaccines in rheumatic patients, including trials, cohorts, cross-sectional and case-control studies involving equal to or more than 10 patients.

### 2.3. Data Extraction

Information regarding humoral and/or cellular immunogenicity, efficacy/effectiveness, local/systemic adverse effects, and disease flares after COVID-19 vaccination were recorded for each study in a standardized Excel file. The influence of relevant drugs, including corticosteroids, conventional synthetic disease-modifying anti-rheumatic drugs (csDMARDs), biologic DMARDs (bDMARDs), targeted synthetic DMARDs (tsDMARDs) such as Janus kinase inhibitors (JAKi), and other immunosuppresants, were also documented.

### 2.4. Statistical Analysis

A statistical analysis was performed using Stata, version 14.0 (StataCorp, College Station, TX, USA). A summary estimate of proportions was derived using the command “metaprop” in a random-effects model. The confidence interval was based on the binomial distribution. The summary estimates of rate ratios (RRs) between rheumatic patients and healthy controls, and between drug users and non-users, were derived using the “metan” command. A random-effects model was used following the procedure of DerSimonian and Laird [23]. The heterogeneity was quantified using Tau^2^, Chi^2^, and I^2^ measures, based on the Mantel-Haenszel model. Begg’s and Egger’s tests were used to assess publication bias, while funnel plots were constructed to visualize asymmetry with respect to immunogenicity and disease flares after COVID-19 vaccination.

## 3. Results

### 3.1. Study Characteristics

The characteristics of the selected studies are demonstrated in Table 1. Most studies were conducted in Western countries and involved Caucasians. In most studies, female adults were predominant, and only one study focused on adolescents [24]. Most study participants received the BNT162b2 vaccine and only a few studies reported on viral vector-based or inactivated vaccines. In terms of rheumatic diseases, inflammatory arthritis and RA in particular constituted the majority of participants in most studies. Twenty-five studies investigated humoral immunogenicity, whereas cellular immunogenicity was less studied, with only seven relevant studies available. Twenty-seven studies documented adverse events based on either clinical records or questionnaires.

### 3.2. Immunogenicity of COVID-19 Vaccines in Rheumatic Patients

**Figure 2:**
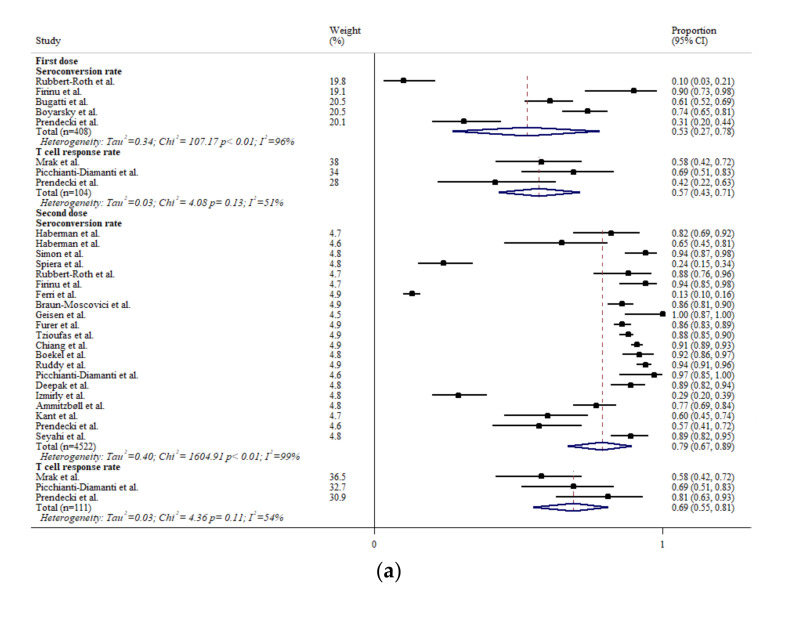

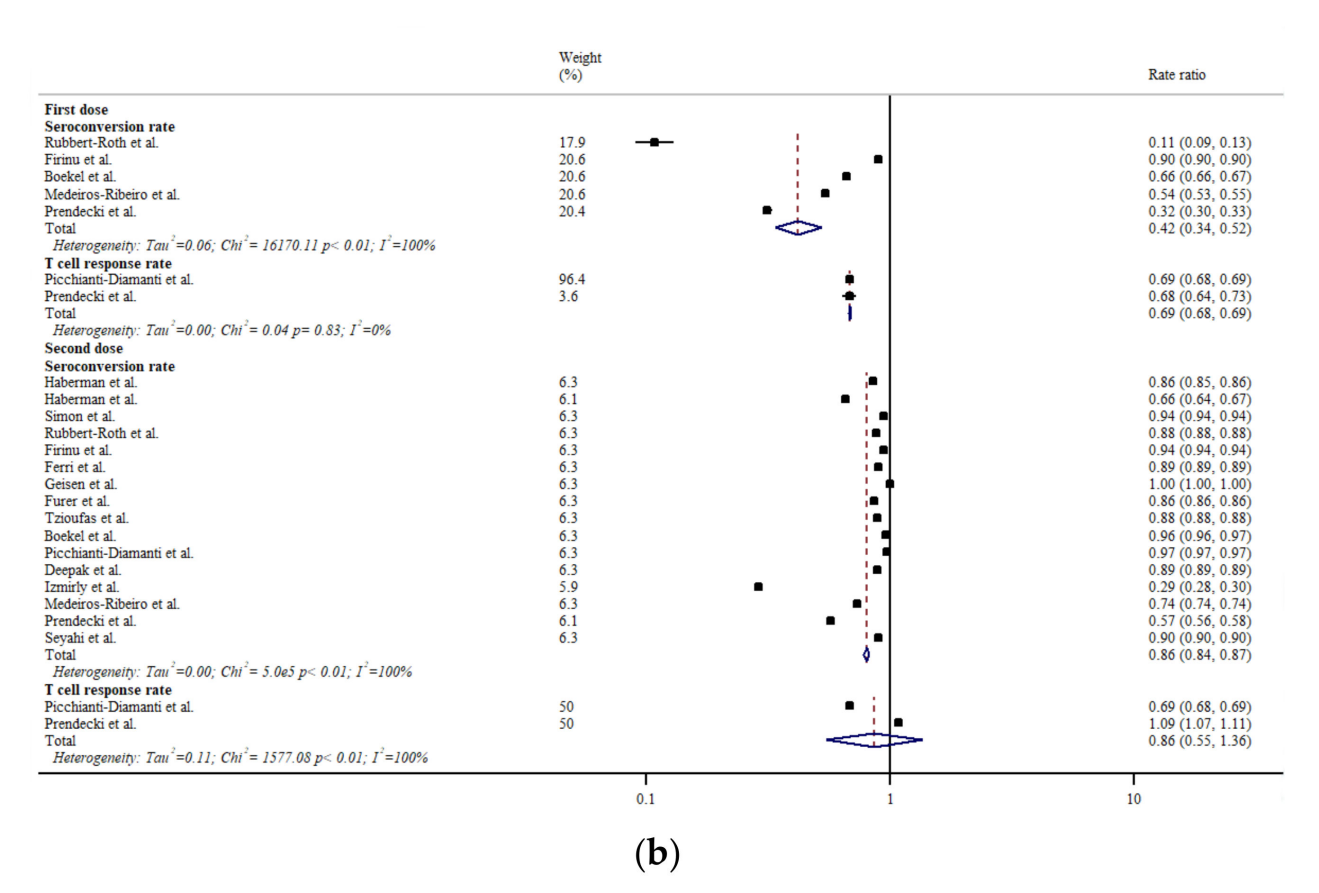
(**a**) The immunogenicity of mRNA vaccines in rheumatic patients, and (**b**) the rate ratios of immunogenicity between rheumatic patients and healthy controls. The black squares represent the effect estimates of the individual studies and the diamonds represent the summary effect estimates.

Humoral response (seroconversion) and T cell response rates after mRNA-based vaccination in rheumatic patients are summarized in Figure 2a. Only 53 (95%CI: 27, 78)% achieved seroconversion after the first dose, although the proportion increased to 79 (95%CI: 67, 89)% after the second dose. The T cell response rate was 57 (95%CI: 43, 71)% after the first dose and, similarly, increased to 69 (95%CI: 55, 81)% after the second dose. Few studies have demonstrated immunogenicity after receiving other vaccines. The seroconversion rate after the first dose of AZD1222 was 49 (95%CI: 44, 54)%, and those after the first and second doses of CoronaVac were 19 (95%CI: 16, 22)% and 70 (67, 73)% respectively [12,31]. The seroconversion rate after Ad26.COV2.S was 80 (95% CI: 65, 90)% [36]. Notably, Schmiedeberg et al. found that a third dose of mRNA vaccine led to a seroconversion rate of 88% (15/17) in RA patients who had no or minimal serological response after two doses [59]. The RRs between rheumatic patients and healthy controls in terms of immunogenicity after COVID-19 vaccination are illustrated in Figure 2b. The RRs for the seroconversion after the first and second dose were 0.42 (95%CI: 0.34, 0.52) and 0.86 (95%CI: 0.84, 0.87), respectively. The RRs for the T cell response after the first and second dose were 0.69 (95%CI: 0.68, 0.69) and 0.86 (95%CI: 0.55, 1.36), respectively. It is worth noting that seroconversion was absent in a few patients receiving anti-CD20 therapy, whereas T cell response could still be elicited in these patients [28,52].

### 3.3. Influencing Factors of Immunogenicity after COVID-19 Vaccination in Rheumatic Patients

**Figure 3:**
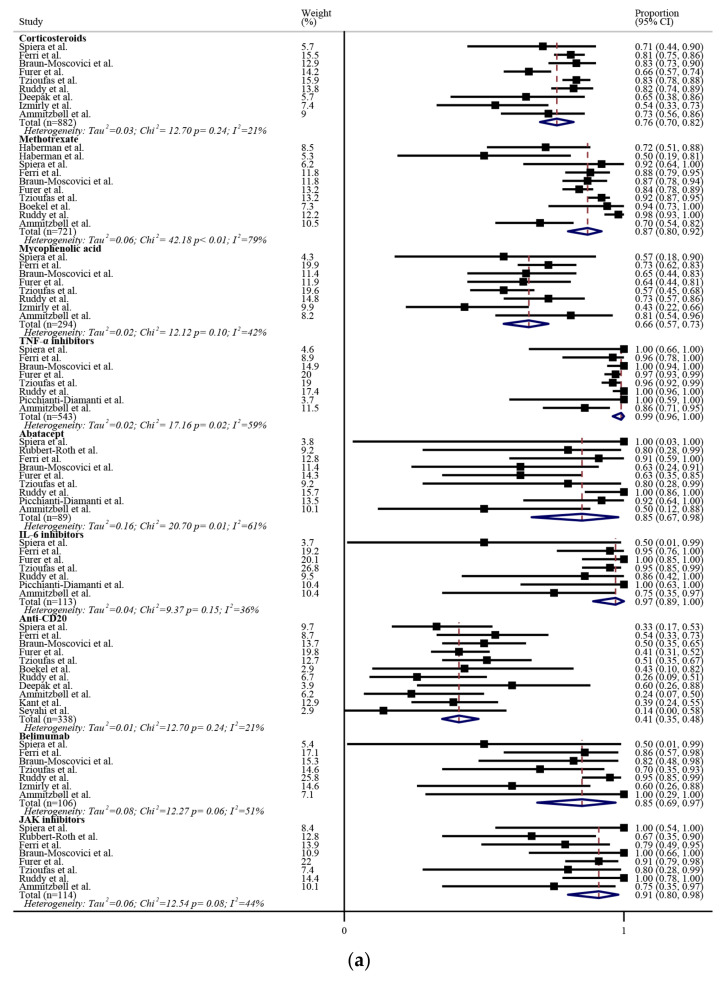

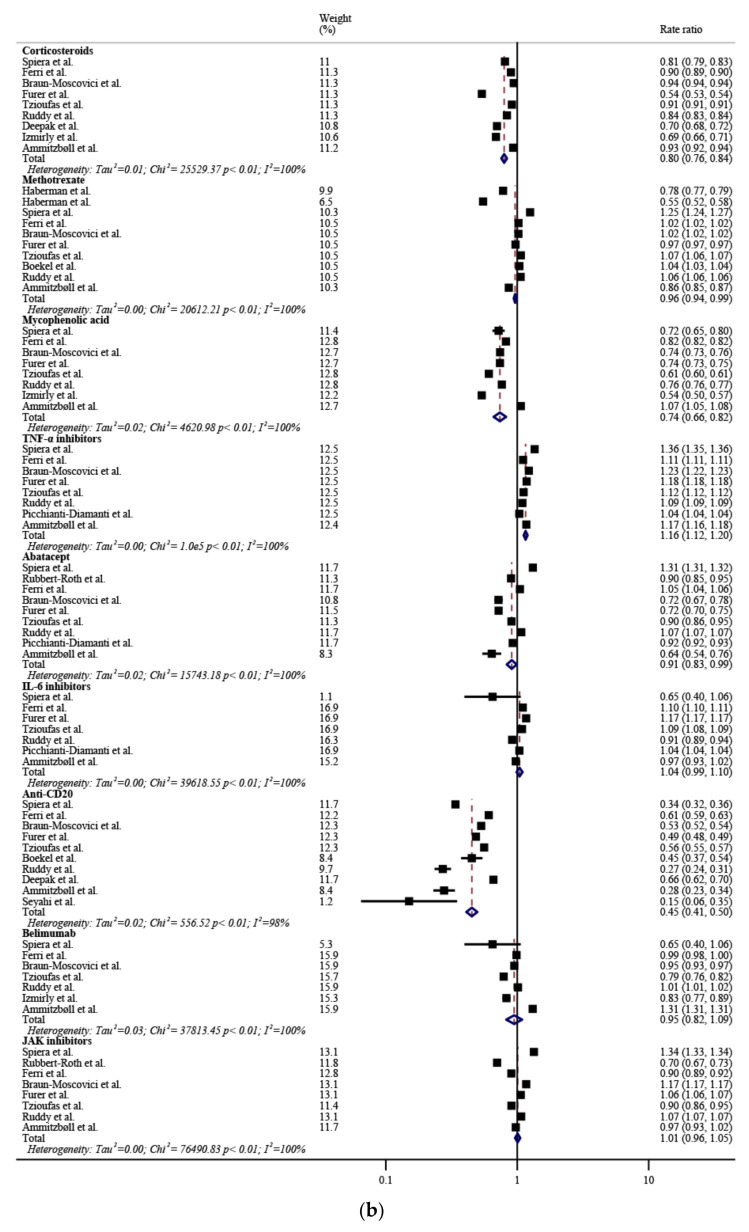
(**a**) Seroconversion rates after the second dose of mRNA vaccines in certain medication users, and (**b**) the seroconversion rate ratios between medication users and non-users in rheumatic patients. The black squares represent the effect estimates of the individual studies and the diamonds represent the summary effect estimates. IL, interleukin; JAK, Janus kinase; TNF, tumor necrosis factor.

The seroconversion rates after the second dose of mRNA vaccines in certain medication users are illustrated in Figure 3a. The use of mycophenolic acid or anti-CD20 therapy was associated with a lower seroconversion rate, i.e., 66 (95%CI: 57, 73)% and 41 (95%CI: 35, 48)%, respectively. The seroconversion rate ratios between medication users and non-users among rheumatic patients after the second dose of mRNA vaccines are illustrated in Figure 3b. The use of corticosteroids, mycophenolic acid, or anti-CD20 therapy was associated with a lower seroconversion rate when compared with non-users. In particular, anti-CD20 therapy was associated with a seroconversion rate that was 55% lower than that of non-users. Additionally, some other factors were found to be associated with a lower seroconversion rate, as summarized in Table 2. To be noted, Bugatti et al. found that withholding methotrexate or b/tsDMARD for a short time did not significantly influence the seroconversion rate [34].

### 3.4. Effectiveness of COVID-19 Vaccines in Rheumatic Patients

Only two retrospective studies demonstrated the effectiveness of COVID-19 vaccines in rheumatic patients. Papagoras et al. showed that the hospitalization and mortality rates were higher in unvaccinated (29% and 4%) than the fully vaccinated rheumatic patients (10% and 0%) [53]. In another study, the effectiveness of two doses of mRNA vaccines against COVID-19 hospitalization was 81%, which was slightly lower than that (90%) of immunocompetent controls [41].

### 3.5. Adverse Events of COVID-19 Vaccines in Rheumatic Patients

**Figure 4:**
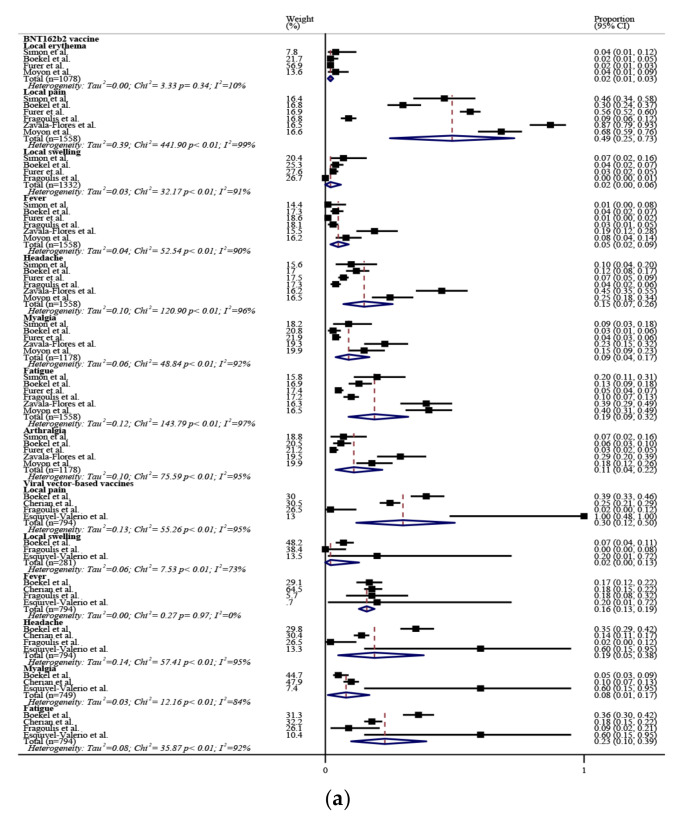

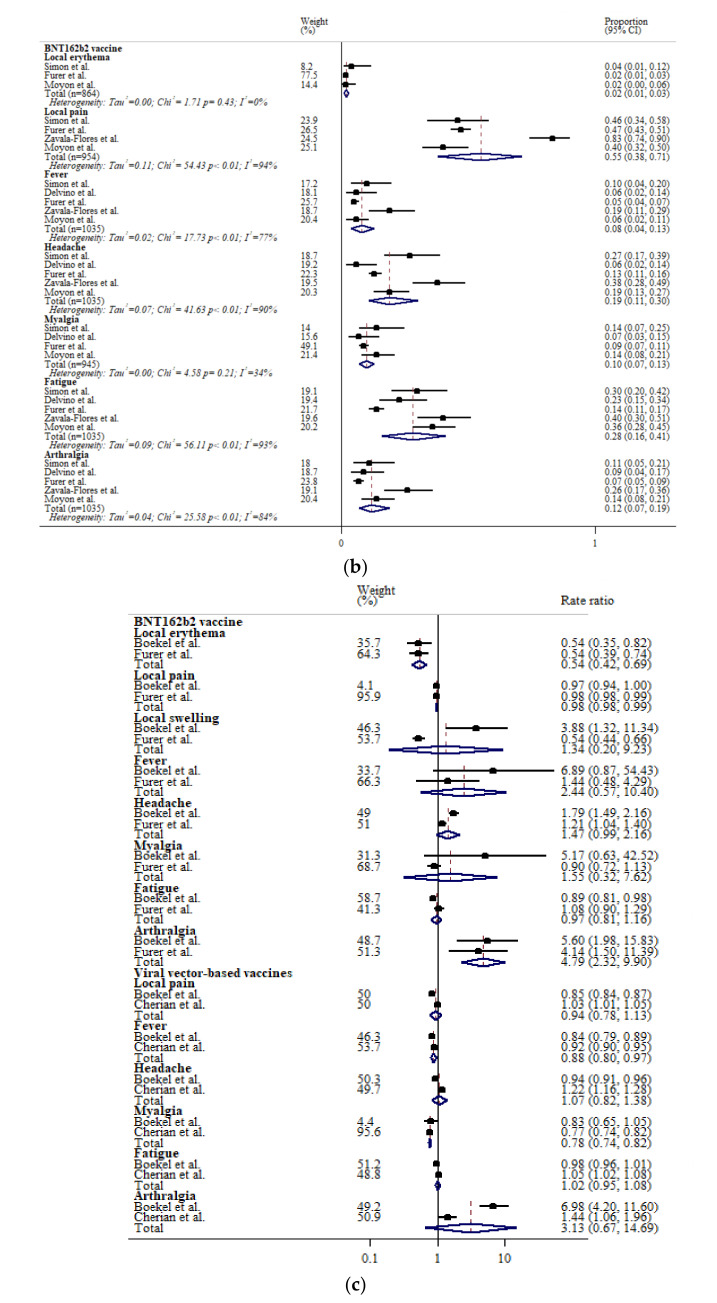
The incidence rate of adverse events after the (**a**) first and (**b**) second dose of COVID-19 vaccines in rheumatic patients, and the (**c**) incidence rate ratios of adverse events after the first dose of COVID-19 vaccines between rheumatic patients and healthy controls. The black squares represent the effect estimates of the individual studies and the diamonds represent the summary effect estimates.

The incidence rates of adverse events after COVID-19 vaccination are illustrated in Figure 4a, b. Local pain (30-55%) was the most common, followed by fatigue (19-28%) after the first dose of viral vector-based vaccine or both doses of the BNT162b2 vaccine. A total of two (0.04%) serious adverse events developed in 4433 rheumatic patients after COVID-19 vaccination. The incidence rate ratios of adverse events after the first dose of COVID-19 vaccines between rheumatic patients and healthy controls are illustrated in Figure 4c. Compared with healthy controls, local pain was less common, whereas arthralgia was more common after receiving the BNT162b2 vaccine, and fever and myalgia were less frequent after viral vector-based vaccination in rheumatic patients.

### 3.6. The Influence of COVID-19 Vaccines on Disease Activity of Rheumatic Diseases

**Figure 5:**
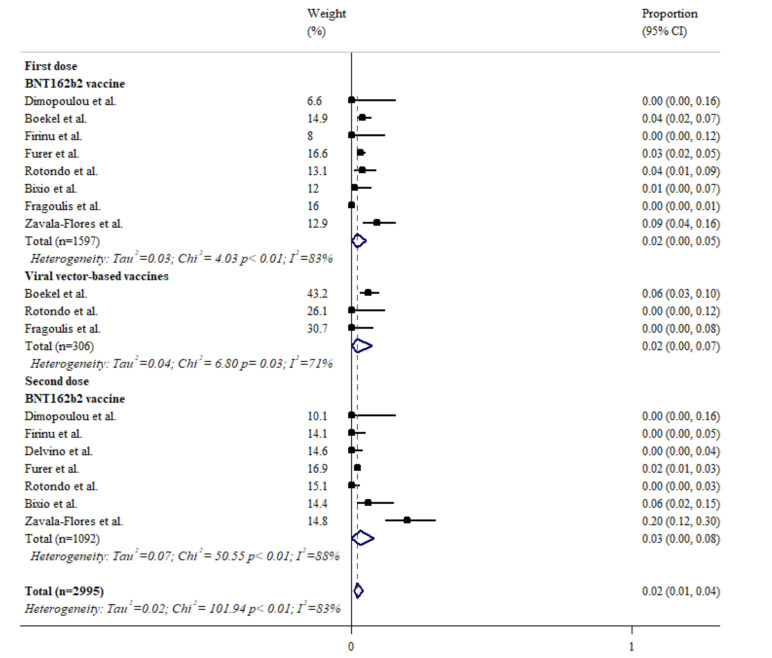
The incidence rate of disease flares after COVID vaccination. The black squares represent the effect estimates of the individual studies and the diamonds represent the summary effect estimates.

As illustrated in Figure 5, around 2–3% of rheumatic patients developed a flare after COVID-19 vaccination. The disease activity measures, such as disease activity score (DAS)28 and Systemic Lupus Disease Activity Index (SLEDAI), etc., were not different before and after the vaccination [13,33,51].

### 3.7. Publication Bias

Visual inspection of funnel plots demonstrated the existence of potential publication biases with regards to seroconversion rate and rate ratios after mRNA vaccines and the proportion of disease flares after vaccination, although the Begg’s and Egger’s test results did not reach statistical significance (Supplementary Figures S1 and S2).

## 4. Discussion

Efficacious COVID-19 vaccination is needed to contain the ongoing pandemic. However, a comprehensive evidence analysis or consensus regarding the efficacy and safety of COVID-19 vaccines in rheumatic patients is lacking to date. According to our review, despite the impaired immunogenicity of vaccines in these patients, the vaccines were still very effective in reducing hospitalization and mortality. There were no new safety signals for these vaccines in rheumatic patients except for arthralgia. In addition, the risk of a disease flare after vaccination was minimal.

Being immunocompromised due to inherent immune dysregulation and the concomitant use of immunosuppressants, rheumatic patients are more vulnerable to severe or opportunistic infections. During the COVID-19 pandemic, rheumatic patients were more likely to be hospitalized or succumb [15,66], and were strongly advised to receive COVID-19 vaccination [67]. We found that rheumatic patients had impaired humoral and cellular immune responses after COVID-19 vaccination, which was consistent with previous observations of poorer responses to different kinds of vaccines [68]. However, the second or even third dose seemed to significantly enhance the immunogenicity of COVID-19 vaccines. Furthermore, retrospective studies revealed that vaccination was still highly effective against severe illness when contracting COVID-19 for rheumatic patients, supporting the importance of full vaccination.

Immunosuppressants and bDMARDs, including methotrexate, abatacept, and anti-CD20 therapy (rituximab), have been shown to impair vaccine response in rheumatic patients [68,69]. In the present meta-analysis, we found impaired humoral response in patients receiving anti-CD20 therapy. In addition, several studies demonstrated that other B lymphocyte-associated factors were also implicated in poor humoral response after COVID-19 vaccination. Therefore, it is better to postpone anti-CD20 therapy until B lymphocytes reconstitution before COVID-19 vaccination. On the other hand, the use of mycophenolic acid was also associated with impaired humoral response after COVID-19 vaccination, which was similarly found in transplantation patients [70,71]. Although withholding most of these immunosuppressants before vaccination was generally recommended [67], we did not find sufficient evidence to support the beneficial effect of such measures, probably due to very few related study results. The only exception was anti-CD20 therapy, in which the interval between the last infusion and COVID-19 vaccination was positively associated with increased humoral response. More studies are required to provide evidence in terms of deciding which medications to withhold and the optimal time frame.

Vaccine hesitancy has led some rheumatic patients to abstain from vaccination, putting them at an unnecessary risk for COVID-19. The two main reasons for hesitation were adverse events and worries about flares of underlying diseases [72]. Our review seeks to reassure such individuals that no new safety signal has been found in rheumatic patients receiving vaccination except for arthralgia after receiving the BNT162b2 vaccine, and most of these joint symptoms were transient and lasted for a few days. The rate of serious adverse events was extremely low after COVID-19 vaccination, i.e., comparable to that in the general population [73]. Additionally, the incidence rate of disease flare after vaccination was also low, and there was, on average, no increase in disease activity in most rheumatic patients.

Our review has some limitations. First, the study population, comorbidities, concomitant medication use, interval between vaccination and outcomes, and outcome measurements differed considerably among studies. Such heterogeneity limited the strength of the interpretation of the results. Furthermore, the combination of several medications precluded the precise determination of the effect of a single drug in terms of immunogenicity. Second, underrepresentation of ethnic groups such as Asians and Hispanics, age groups such as adolescents and children, receivers of non-mRNA vaccines, and patients with rheumatic diseases other than inflammatory arthritis should cause some concern when extrapolating our findings. Third, we found that the humoral immune response was more impaired than the cellular response in rheumatic patients after COVID-19 vaccination. Could such preserved cellular response protect these patients from COVID-19 infection? A relatively small number of effectiveness studies suggested an urgent need to conduct more such studies to elucidate this question and find other influencing factors (such as medications). Fourth, the duration of medication use was not specified in these studies. For instance, the duration of corticosteroid use may affect vaccine efficacy. Despite these limitations, our review still provides an updated and valuable overview of effectiveness and safety issues while rheumatic patients worldwide are receiving vaccinations due to the COVID-19 pandemic. Moreover, our results were similar to those of studies on patients with multiple sclerosis, an autoimmune neurological disorder which is treated with similar drugs [74,75].

## 5. Conclusions

Our comprehensive review demonstrated the efficacy, albeit lower when compared with healthy individuals, and safety of COVID-19 vaccines in rheumatic patients. The results support the recommendations of full vaccination in these patients. However, significant study heterogeneity may undermine our conclusions.

## Figures and Tables

**Table 1:** Study characteristics.

Author	Country	Sample Size of Rheumatic Patients (*n*)	Proportion of Female	Mean/Median Age (Years)	Vaccine	Rheumatic Diseases	Humoral Immunogenecity Measurement	Cellular Immunogenecity Measurement	Documentation of Adverse Events
Ammitzbøll et al. [25]	Denmark	134	72%	66	BNT	RA 54%, and SLE 46%	Anti-SARS-CoV-2 antibody CLIA (Ortho Clinical Diagnostics)	N.A.	N.A.
Barbhaiya et al. [26]	USA	1101	81%	61	BNT 54%, Moderna 44%, J&J 2%, and AZ 0.3%	N.A.	N.A.	N.A.	Online survey
Bartels et al. [27]	Denmark	282	79%	59	BNT	RA 55%, and SLE 45%	N.A.	N.A.	Questionnaire
Benucci et al. [28]	Italy	14	N.A.	57	BNT	RA	Anti-RBD IgG antibodies FEIA (ThermoFisher)	IGRA (Euroimmun)	N.A.
Bixio et al. [29]	Italy	77	81%	62	BNT	RA	N.A.	N.A.	Clinical record
Boekel et al. [30]	Netherlands	505	65%	64	AZ 46%, BNT 41%, and Moderna 13%	RA 40%, PsA 10%, and MS 16%	N.A.	N.A.	Online questionnaire
Boekel et al. [31]	Netherlands	632	67%	63	AZ 54%, BNT 38%, and Moderna 8%	RA 41%, PsA 11%, AS 11%, and MS 9%	Anti-RBD IgG antibody ELISA (in-house)	N.A.	N.A.
Boyarsky et al. [32]	USA	123	95%	50	BNT 52%, and Moderna 48%	Inflammatory arthritis 28%, overlap syndrome 29%, SLE 20%, and PSS 13%	Anti-RBD antibody ECLIA (Roche)	N.A.	N.A.
Braun-Moscovici et al. [33]	Isreal	264	76%	58	BNT	RA 37%, PsA 12%, and SpA 8%	Anti-RBD IgG CLIA (Abbott)	N.A.	Clinical record
Bugatti et al. [34]	Italy	140	68%	56	BNT	RA 59%, PsA 21%, and SpA 20%	Anti-S1/S2 protein antibodys IgG CLIA (DiaSorin)	N.A.	N.A.
Cherian et al. [35]	India	513	83%	58	AZ 87%, and Covaxin 10%	RA 44%, inflammatory arthritis 16%, SpA 13%, and SLE 10%,	N.A.	N.A.	Clinical record
Chiang et al. [36]	USA	1039	94%	46	mRNA vaccines 96%, and J&J 4%	Inflammatory arthritis 44%, overlap syndrome 21%, SLE 21%, and PSS 5%	Anti-RBD antibody ECLIA (Roche)	N.A.	N.A.
Connolly et al. [37]	USA	1377	92%	47	BNT 55%, and Moderna 45%	Inflammatory arthriitis 47%, SLE 20%, and overlap syndrome 20%	N.A.	N.A.	Online questionnaire
Cuomo et al. [38]	Italy	27	78%	49	BNT	Inflammaory arthritis 48%, RA 22%, and SSc 19%	N.A.	N.A.	Telephone interview
Deepak et al. [39]	USA	133	74%	46	mRNA vaccines	IBD 32%, RA 29%, SpA 15%, and SLE 11%	Anti-S protein IgG ELISA (in-house)	N.A.	N.A.
Delvino et al. [40]	Italy	81	68%	76	BNT	GCA	N.A.	N.A.	Written questionnaire
Dimopoulou et al. [24]	Greece	21	76%	17	BNT	JIA	N.A.	N.A.	N.A.
Embi et al. [41]	USA	5024	N.A.	N.A.	Moderna 40%, and BNT 60%	Rheumatic or inflammatory disorders	N.A.	N.A.	N.A.
Esquivel-Valerio et al. [42]	Mexico	225	95%	51	BNT 48%, Convidecia 13%, Moderna 13%, AZ 12%, CoronaVac 10%, and J&J 2%	RA 59%, SLE 11%, and axial SpA 10%	N.A.	N.A.	Survey
Ferri et al. [43]	Italy	478	84%	59	BNT 94%, and Moderna 6%	SSc 55%, RA 21%, CV 13%, and SLE 8%,	Anti- S1/S2 protein antibodys IgG CLIA (Abbott)	N.A.	Telephone interview
Firinu et al. [44]	Italy	95	73%	56	BNT	SLE 24%, RA 24%, PsA, PsO and AS 25%	Anti-RBD IgG CLIA (Snibe Diagnostics)	N.A.	N.A.
Fragoulis et al. [45]	Greece	441	76%	56	BNT 86%, AZ 10%, Moderna 3%, and J&J 1%	Inflammatory arthritis 59%, CTD 27%, and vasculitis 11%	N.A.	N.A.	Telephone interview
Furer et al. [13]	Isreal	686	69%	59	BNT	RA 38%, PsA 24%, SLE 15%, vasculitis 10%, and SpA 10%	Anti-S1/S2 protein antibodys IgG CLIA (DiaSorin)	N.A.	Telephone questionnaire
Geisen et al. [22]	Germany	26	64%	51	81% BNT, and Moderna 19%	RA 31%, PsO 12%, SpA 12%, and IBD 12%	Anti-SARS-CoV-2 ELISA (Euroimmun)	N.A.	Online survey
Haberman et al. [46]	USA	51	71%	56	BNT	RA 43%, and PsO/PsA 47%	Anti-S1 protein antibody ELISA (in-house)	N.A.	N.A.
	Germany	31	71%	51	BNT	GCA and PMR	Anti-S1 protein antibody ELISA (Euroimmun)	N.A.	N.A.
Izmirly et al. [47]	USA	90	88%	46	BNT 68%, Moderna 15%, and J&J 5.5%	SLE	Anti-RBD IgG ELISA (in-house)	IFN-γ ELISpot assay (in-house)	N.A.
Kant et al. [48]	USA	48	35%	67	Moderna 52%, BNT 40%, and J&J 8%	AAV	N.A.	N.A.	N.A.
Li et al. [49]	Hong Kong	1324	75%	58	CoronaVac 51%, and BNT 49%,	RA	N.A.	N.A.	Clinical record
Machado et al. [50]	EULAR COVID-19 Vaccination Registry	1519	68%	63	BNT 78%, AZ 16%, and Moderna 5%	Inflammatory arthritis 51%, CTD 19%, and vasculitis 16%	N.A.	N.A.	Clinical record
Medeiros-Ribeiro et al. [12]	Brazil	910	77%	51	CoronaVac	Inflammatory arthritis 50%	Anti-S1/S2 protein antibodys IgG CLIA (DiaSorin)	N.A.	Diary
Moyon et al. [51]	France	126	91%	47	BNT	SLE	SARS-CoV-2 multi-antigenphotonic ring immunoassay(Genalyte)	IGRA (Qiagen)	Clinical record
Mrak et al. [52]	Austria	45	78%	64	BNT 82%, and Moderna 18%	RA 53%, CTD 27%, and vasculitis 16%	N.A.	IFN-γ ELISpot assay (in-house)	N.A.
Papagoras et al. [53]	Greece	48	69%	51	BNT 79%, and AZ 21%	Inflammatory arthritis 58%, CTD and vasculitis 40%	N.A.	N.A.	N.A.
Picchianti-Diamanti et al. [54]	Italy	35	77%	59	BNT	RA	Anti-RBD IgG CLIA (Abbott)	IFN-γ whole-blood assay (in-house)	N.A.
Prendecki et al. [19]	UK	119	48%	53	mRNA vaccines 71%, and AZ 29%,	AAV/anti-GBM 38%, MCD/FSGS 24%, MGN 19%, and SLE 16%	Anti-S1/S2 protein antibodys IgG CLIA (Abbott)	T SPOT (Oxford Immunotec)	N.A.
Rotondo et al. [55]	Italy	137	70%	57	BNT 78%, and AZ 22%	Arthritis 78%, and CTD 18%	N.A.	N.A.	Questionnaire
Rubbert-Roth et al. [56]	Switzerland	53	55%	65	BNT 83%, and Moderna 17%	RA	Anti-RBD antibody ECLIA (Roche)	N.A.	N.A.
Ruddy et al. [57]	USA	404	96%	44	Moderna 51%, and BNT 49%	Inflammatory arthritis 45%, and SLE 22%	Anti-RBD antibody ECLIA (Roche)	N.A.	N.A.
Sattui et al. [58]	Global RheumatologyAlliance	2860	87%	55	BNT 53%, AZ 23%, Moderna 21%, and J&J 2%	RA 42%, IIM 17%, PSS 15%, and SLE 14%	N.A.	N.A.	Online survey
Schmiedeberg et al. [59]	Switzerland	17	47%	67	BNT 82%, and Moderna 12%	RA	Anti-RBD antibody ECLIA (Roche)	N.A.	N.A.
Sciascia et al. [60]	Italy	102	85%	52	BNT 66%, and Moderna 34%	APS 51%, and aPL 49%	N.A.	N.A.	Clinical record
Seyahi et al. [61]	Turkey	104	66%	48	CoronaVac	SpA 23%, RA 18%, CTD 16%, BS 14%, and FMF 10%	Anti-RBD antibody ECLIA (Roche)	N.A.	N.A.
Simon et al. [62]	Germany	84	66%	53	BNT	SpA 32%, RA 30%, IBD 10%, and PsO 10%	Anti-S1 protein antibody ELISA (Euroimmun)	N.A.	Clinical record
Spiera et al. [59]	USA	89	76%	61	BNT 57%, and Moderna 43%	RA 26%, GPA 13%, PSS 11%, and SLE 10%	Anti-RBD antibody ECLIA (Roche)	N.A.	N.A.
Tzioufas et al. [63]	Greece	605	71%	58	BNT 95%, and Moderna 5%	RA 28%, seronegative arthritis 21%, SLE 20%, and vasculitis 11%,	Anti-S1 protein antibody ELISA (Euroimmun)	N.A.	Questionnaire
Yang et al. [64]	USA	70	69%	48	mRNA vaccines	RA 30%, SpA 30%, SLE 11%, and IBD 10%	N.A.	N.A.	Clinical record
Zavala-Flores et al. [65]	Peru	100	94%	39	BNT	SLE	N.A.	N.A.	Clinical record

AAV, antineutrophil cytoplasmic antibody-associated vasculitis; aPL, antiphospholipid antibodies positivity; APS, antiphospholipid syndrome; AS, ankylosing spondylitis; AZ, AZD1222; BNT, BNT162b2; BS, Behcet’s syndrome; CLIA, chemiluminescent immunoassay; CTD, connective tissue disorder; CV, cryoglobulinemic vasculitis; ECLIA, electrochemiluminescence immunoassay; ELISA, enzyme-linked immunosorbent assay; ELISpot, Enzyme-linked immunospot; FEIA, fluorescent enzyme immunoassay; FMF, familial mediterranean fever; FSGS, focal segmental glomerulosclerosis; GBM, glomerular basement membrane; GCA, giant cell arteritis; GPA, granulomatosis with polyangiitis; IBD, inflammatory bowel disease; IFN, interferon; IGRA, interferon-γ release assay; IIM, idiopathic inflammatory myositis; J&J, Ad26.COV2.S.; JIA, juvenile idiopathic arthritis; MCD, minimal change disease; MGN, membranous glomerulonephritis; Moderna, mRNA-1273; MS, multiple sclerosis; N.A., not available; PMR, polymyalgia rheumatica; PsA, psoriatic arthritis; PsO, psoriasis; PSS, primary Sjogren’s syndrome; RA, rheumatoid arthritis; RBD, receptor-binding domain; SLE, systemic lupurs erythematosus; SpA, spondyloarthropathy; SSc, systemic sclerosis.

**Table 2:** Factors other than medication use that were associated with a lower seroconversion rate after COVID-19 vaccination in rheumatic patients.

Factors
Older age
Lower B lymphocyte count
Lower serum IgG
Shorter interval between vaccination and last infusion of anti-CD20 therapy
Not achieving B cell reconstitution after anti-CD20 therapy

## Data Availability

The datasets used and/or analyzed during the current review are available from the corresponding author on reasonable request.

## Supplementary Materials

The following are available online at https://www.mdpi.com/article/10.3390/biomedicines10040834/s1, Figure S1: The funnel plots, as well as Begg’s and Egger’s tests results, of the seroconversion rate and cellular response rate after (a, b) 1st and (c, d) 2nd dose of mRNA vaccines, and (e) the proportion of disease flares after COVID-19 vaccination, Figure S2: Begg’s and Egger’s tests results, of the seroconversion rate ratios after (a) 1st and (b) 2nd dose of mRNA vaccines when compared with healthy controls, Table S1: Search strategies.
