# Supplementary material for: Immunogenicity, Effectiveness, and Safety of COVID-19 Vaccines in Rheumatic Patients: An Updated Systematic Review and Meta-Analysis
Source: Biomedicines. 2022 Apr 1;10(4):834. doi: 10.3390/biomedicines10040834 (PMC9030402; doi:10.3390/biomedicines10040834)
Supplement: Supplementary file 1 [file biomedicines-10-00834-s001.zip › biomedicines-1612250-supplementary.pdf]

Table S1. Search strategies.

PubMed	
#1	COVID-19 Vaccines[MeSH] OR COVID 19 Vaccine*[tiab] OR COVID-19 Virus Vaccine*[tiab] OR COVID-19 Vaccine*[tiab] OR COVID 19 Virus Vaccine*[tiab] OR COVID19 Vaccine*[tiab] OR SARS-CoV-2 Vaccine*[tiab] OR SARS CoV 2 Vaccine*[tiab] OR SARS2 Vaccine*[tiab] OR Coronavirus Disease 2019 Vaccine*[tiab] OR Coronavirus Disease 2019 Virus Vaccine*[tiab] OR Coronavirus Disease-19 Vaccine*[tiab] OR Coronavirus Disease 19 Vaccine*[tiab] OR 2019-nCoV Vaccine*[tiab] OR 2019 nCoV Vaccine*[tiab] OR 2019 Novel Coronavirus Vaccine*[tiab] OR SARS Coronavirus 2 Vaccine*[tiab] OR mRNA-1273 vaccine[Supplementary Concept] OR mRNA-1273*[tiab] OR Moderna*[tiab] OR ChAdOx1 COVID-19 vaccine[Supplementary Concept] OR ChAdOx1*[tiab] OR astrazeneca*[tiab] OR AZD1222*[tiab] OR Ad26.COV2.S vaccine[Supplementary Concept] OR Ad26*[tiab] OR JNJ 78436735*[tiab] OR JNJ-78436735*[tiab] OR BNT162 vaccine[Supplementary Concept] OR BNT*[tiab] OR Pfizer-BioNTech*[tiab] OR Covishield*[tiab] OR Covaxin*[tiab] OR BBV152*[tiab] OR BBIBP-CorV*[tiab] OR Sinopharm*[tiab] OR CoronaVac*[tiab] OR Sinovac*[tiab]
#2	Connective Tissue Diseases[MeSH] OR Connective Tissue*[tiab]
#3	Rheumatic Diseases[MeSH] OR Rheuma*[tiab]
#4	systemic lupus*[tiab] OR Libman Sacks*[tiab]
#5	Arthritis[MeSH] OR arthri*[tiab] OR Polyarthri*[tiab]
#6	Sjogren's Syndrome[MeSH] OR Sjogren*[tiab] OR Sicca*[tiab]
#7	Spondylarthropathies[MeSH] OR Spondyloarthro*[tiab] OR Spondylarthro*[tiab]
#8	Systemic Sclero*[tiab] OR Localized Sclero*[tiab] OR Morphea*[tiab] OR Linear Sclero*[tiab]
#9	Myositis[MeSH] OR Myosit*[tiab] OR Inflammatory Muscle*[tiab] OR Inflammatory Myopath*[tiab] OR Idiopathic Inflammatory Myo*[tiab]
#10	Hughes Syndrome*[tiab] OR Antiphospholipid*[tiab] OR Anti-Phospholipid*[tiab] OR Anti Phospholipid*[tiab]
#11	Vasculitis[MeSH] OR Vasculiti*[tiab] OR Angiiti*[tiab]
#12	Cryoglobulinemia[MeSH] OR Cryoglobulin*[tiab]
#13	Still's Disease, Adult-Onset[MeSH] OR Adult-Onset Still*[tiab] OR Adult Onset Still*[tiab]
#14	Fibromy*[tiab] OR Fibrositi*[tiab] OR Secondary Fibromy*[tiab]
#15	#1 AND (#2 OR #3 OR #4 OR #5 OR #6 OR #7 OR #8 OR #9 OR #10 OR #11 OR #12 OR #13 OR #14) Filters: from 2020/1/1 - 3000/12/12

EMBASE

#1 ‘ SARS-CoV-2 vaccine’/exp OR ‘2019-nCoV vaccine*’:ab,ti,kw OR ‘2019-nCoV virus vaccine*’:ab,ti,kw OR ‘coronavirus disease 2019 vaccine*’:ab,ti,kw OR ‘COVID 19 vaccine*’:ab,ti,kw OR ‘COVID-19 vaccine*’:ab,ti,kw OR ‘COVID-19 virus vaccine*’:ab,ti,kw OR ‘COVID19 vaccine*’:ab,ti,kw OR ‘COVID19 virus vaccine*’:ab,ti,kw OR ‘HCoV-19 vaccine*’:ab,ti,kw OR ‘HCoV-19 virus vaccine*’:ab,ti,kw OR ‘coronavirus 2019 vaccine*’:ab,ti,kw OR ‘SARS-CoV-2 vaccine*’:ab,ti,kw OR ‘nCoV-2019 vaccine*’:ab,ti,kw OR ‘nCoV-2019 virus vaccine*’:ab,ti,kw OR ‘coronavirus vaccine*’:ab,ti,kw OR ‘SARS Coronavirus 2 vaccine*’:ab,ti,kw OR ‘SARS-CoV-2 inactivated vaccine*’:ab,ti,kw OR ‘SARS-CoV-2 virus vaccine*’:ab,ti,kw OR ‘SARS2 vaccine*’:ab,ti,kw OR ‘SARS2 virus vaccine*’:ab,ti,kw OR ‘severe acute respiratory syndrome 2 vaccine*’:ab,ti,kw OR ‘severe acute respiratory syndrome coronavirus 2 vaccine*’:ab,ti,kw OR ‘moderna*’:ab,ti,kw OR ‘RNA-1273*’:ab,ti,kw OR ‘RNA1273*’:ab,ti,kw OR ‘bnt*’:ab,ti,kw OR ‘ad26*’:ab,ti,kw OR ‘janssen*’:ab,ti,kw OR ‘jnj*’:ab,ti,kw OR ‘31518*’:ab,ti,kw OR ‘astrazeneca*’:ab,ti,kw OR ‘azd*’:ab,ti,kw OR ‘chadox*’:ab,ti,kw OR ‘covishield*’:ab,ti,kw OR ‘bbv*’:ab,ti,kw OR ‘covaxin*’:ab,ti,kw OR ‘bbibp*’:ab,ti,kw OR ‘sinopharm*’:ab,ti,kw OR ‘sinovac*’:ab,ti,kw OR ‘coronavac*’:ab,ti,kw

#2 ‘ connective tissue disease’/exp OR ‘connective tissue*’:ab,ti,kw

#3 ‘ rheumatic disease’/exp OR ‘rheuma*’:ab,ti,kw

#4 ‘ systemic lupus*’:ab,ti,kw OR ‘lupus*’:ab,ti,kw

#5 ‘ arthri’:ab,ti,kw OR ‘joint inflammat*’:ab,ti,kw

#6 ‘ Sjogren*’:ab,ti,kw OR ‘sicca*’:ab,ti,kw

#7 ‘ spondylarthropathy’/exp OR ‘spondyl*’:ab,ti,kw

#8 ‘ sclerodem*’:ab,ti,kw OR ’systemic sclera*’:ab,ti,kw

#9 ‘ myositis’/exp OR ‘idiopathic inflammatory myo*’:ab,ti,kw OR ‘inflammatory myo*’:ab,ti,kw OR ‘myositis*’:ab,ti,kw

#10 ‘ Hughes syndrome*’:ab,ti,kw OR ‘antiphospholipid*’:ab,ti,kw OR ‘primary antiphospholipid*’:ab,ti,kw

#11 ‘ vasculitis’/exp OR ‘vasculiti*’:ab,ti,kw OR ‘angiiti*’:ab,ti,kw

#12 ‘ cryoglobulinemia’/exp OR ‘cryoglobulin*’:ab,ti,kw

#13 ‘ adult-Onset Still*’:ab,ti,kw

#14 ‘ fibromyalgia’/exp OR ‘fibrositi*’:ab,ti,kw

#15 #1 AND (#2 OR #3 OR #4 OR #5 OR #6 OR #7 OR #8 OR #9 OR #10 OR #11 OR #12 OR #13 OR #14) AND [2020-2021]/py

(a)

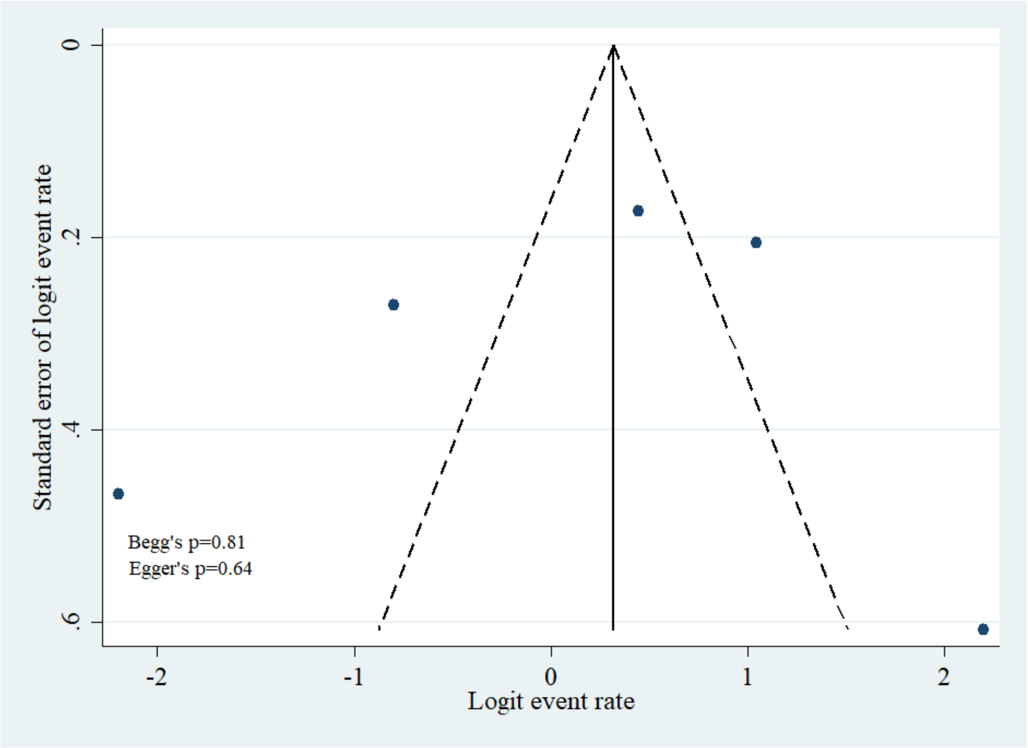
Standard error of logit event rate
0
.2
.4
.6
-2
-1
0
1
2
Logit event rate
Begg's p=0.81
Egger's p=0.64

(b)

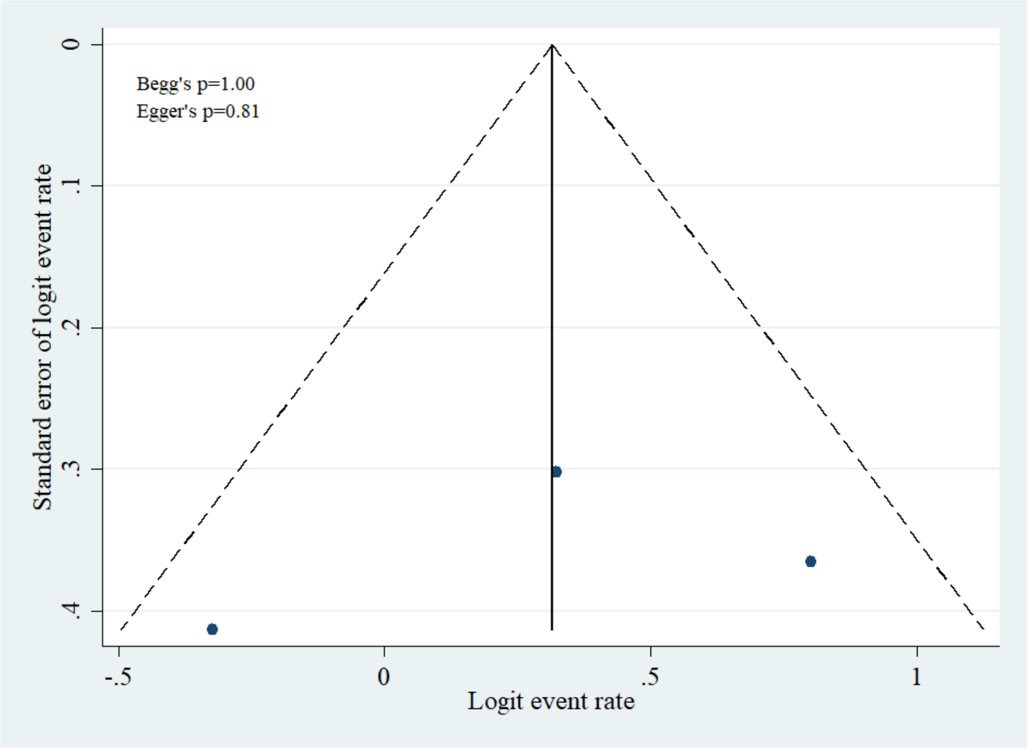
Standard error of logit event rate
0
.1
.2
.3
.4
-.5
0
.5
1
Logit event rate
Begg's p=1.00
Egger's p=0.81

(c)

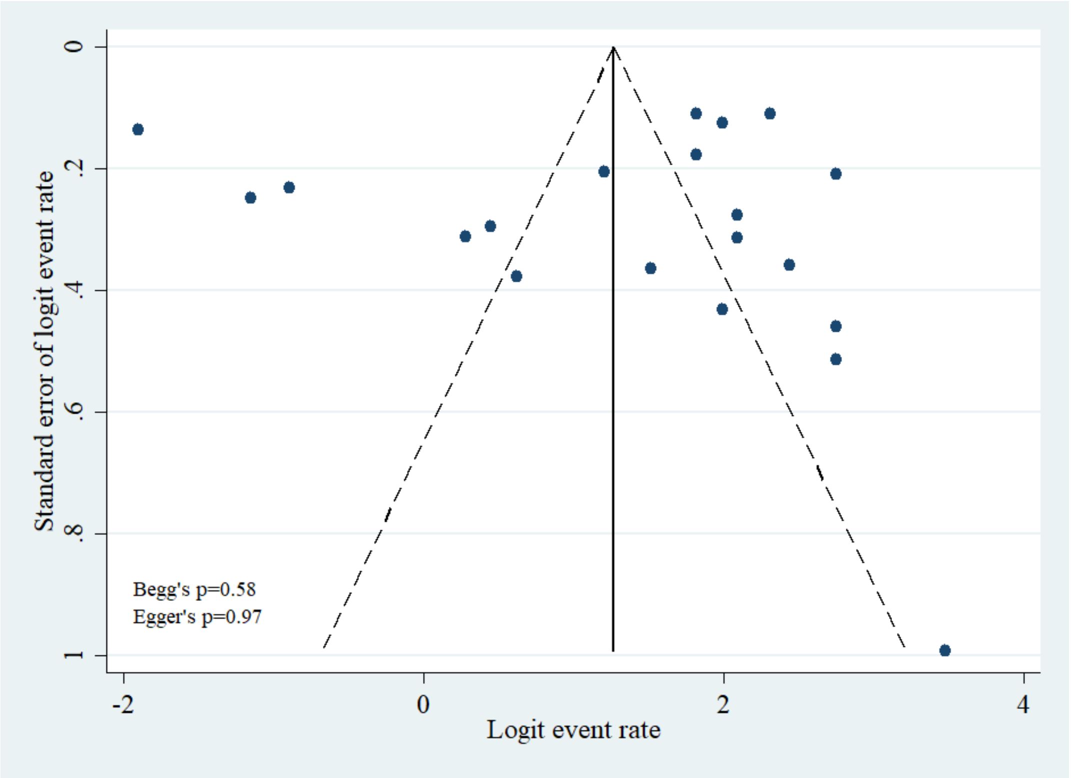

Begg's p=0.58
Egger's p=0.97
Standard error of logit event rate
0
.2
.4
.6
.8
1
-2
0
2
4
Logit event rate

(d)

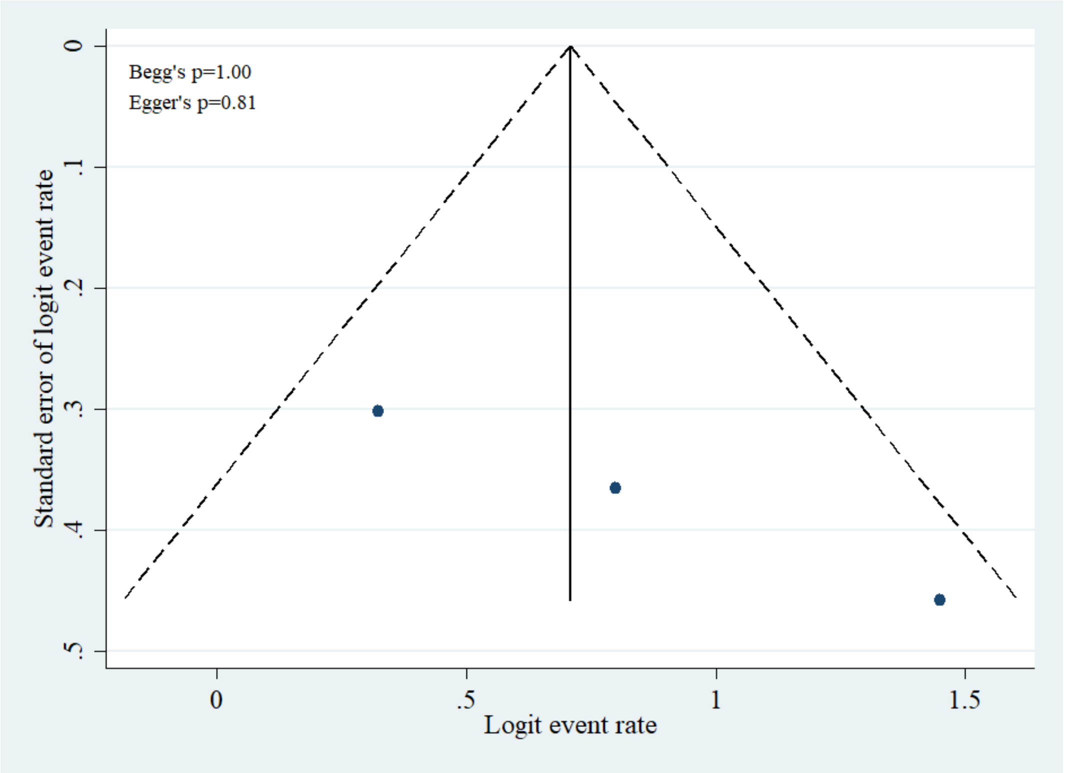

Begg's p=1.00
Egger's p=0.81
Standard error of logit event rate
0
.1
.2
.3
.4
.5
0
.5
1
1.5
Logit event rate

(e)

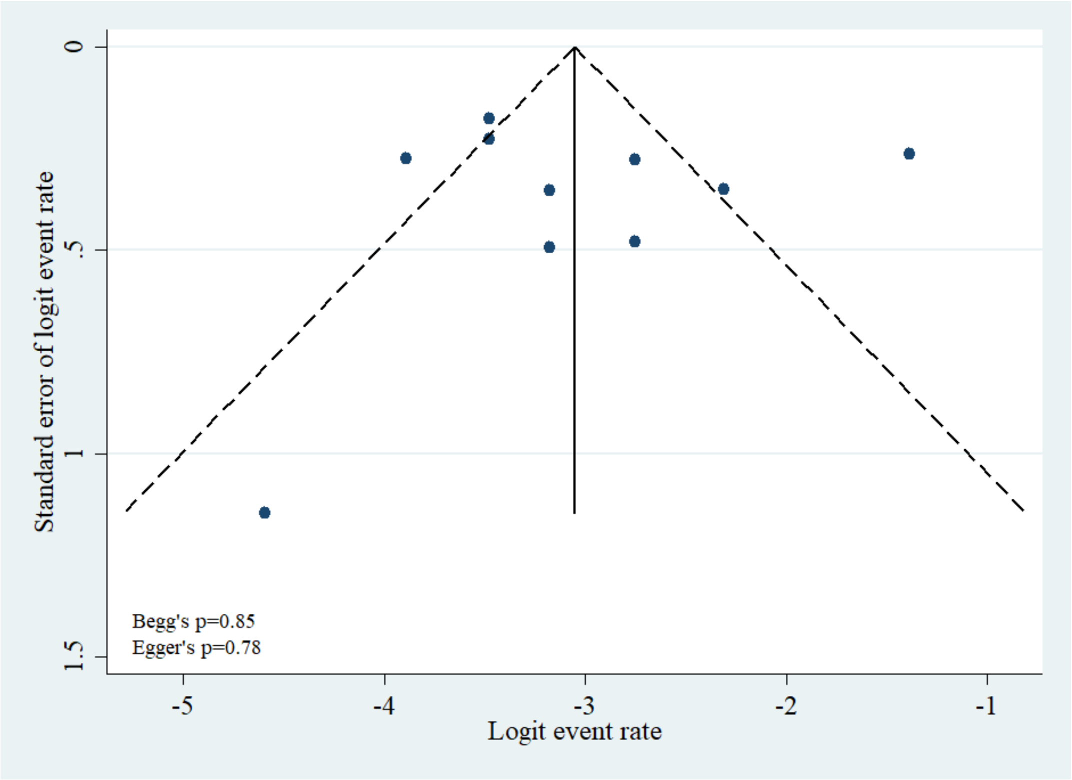

Figure S1. The funnel plots, as well as Begg's and Egger's tests results, of the seroconversion rate and cellular response rate after (a, b) 1st and (c, d) 2nd dose of mRNA vaccines, and (e) the proportion of disease flares after COVID-19 vaccination.

(a)

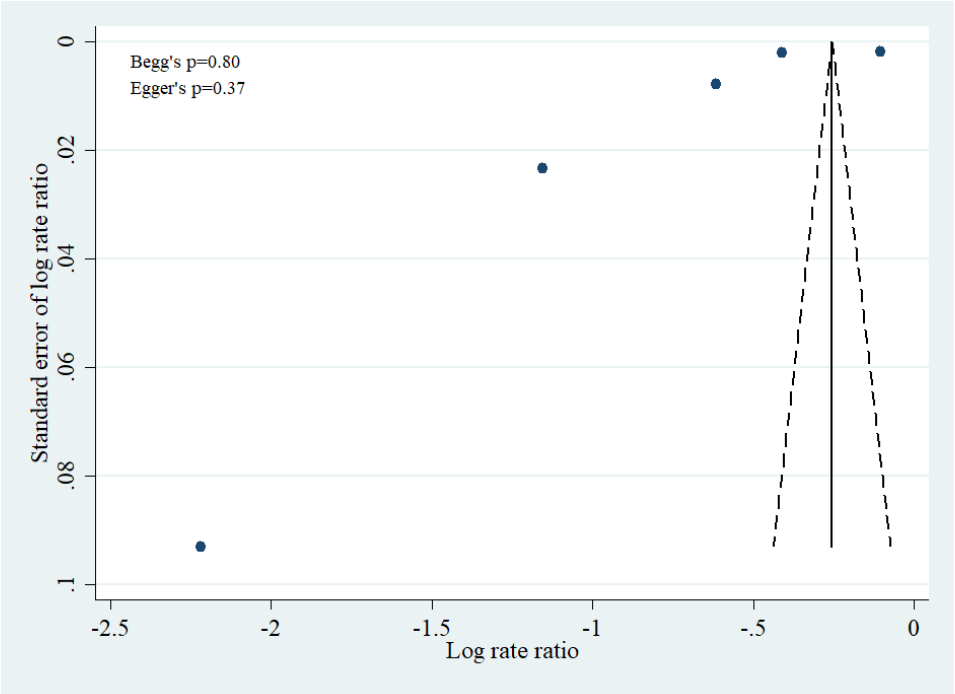

(b)

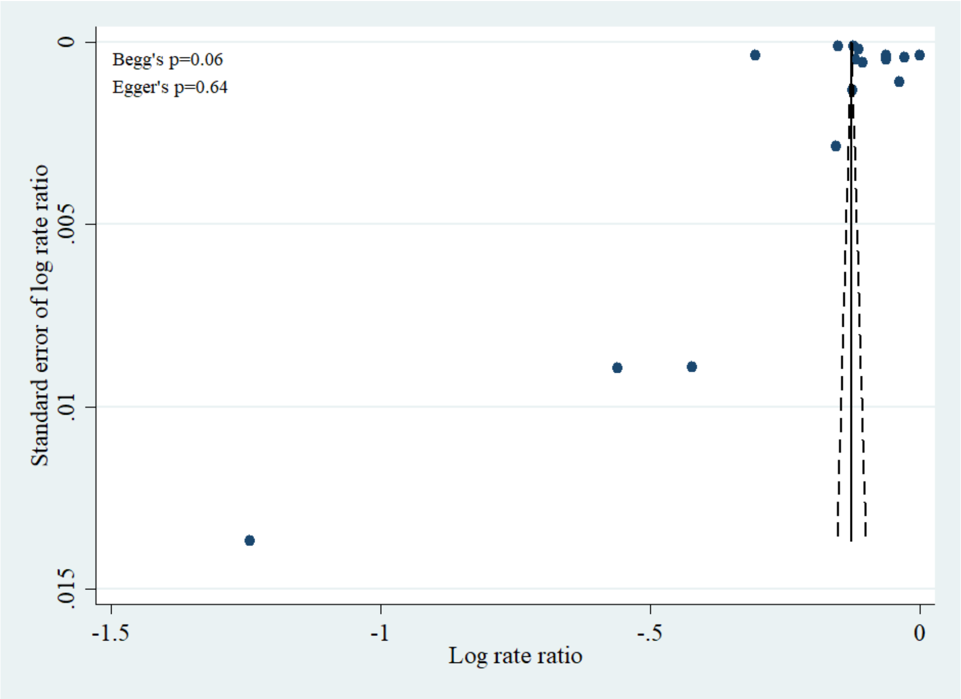

Figure S2. The funnel plots, as well as Begg's and Egger's tests results, of the seroconversion rate ratios after (a) 1st and (b) 2nd dose of mRNA vaccines when compared with healthy controls.
